# Risk factors of acute kidney injury in patients with Stanford type B aortic dissection involving the renal artery who underwent thoracic endovascular aortic repair

**DOI:** 10.1080/0886022X.2021.1949349

**Published:** 2021-07-11

**Authors:** Xiuping An, Xi Guo, Nan Ye, Weijing Bian, Xiaofeng Han, Guoqin Wang, Hong Cheng

**Affiliations:** a Division of Nephrology, Beijing AnZhen Hospital, Capital Medical University, Beijing, China; b Division of Diagnostic and Interventional Radiology, Beijing AnZhen Hospital, Capital Medical University, Beijing, China

**Keywords:** Acute kidney injury, type B aortic dissection, TEVAR, risk factors

## Abstract

**Background:**

Acute kidney injury (AKI) is one of the most common and serious complications in patients with type B aortic dissection (TBAD). This study aimed at investigating the incidence and risk factors of in-hospital AKI in TBAD patients involving the renal artery who underwent thoracic endovascular aortic repair (TEVAR) only.

**Methods:**

A total of 256 patients who were diagnosed as TBAD combined with renal artery involvement were included in this retrospective study. All patients were divided into the AKI group and the non-AKI group according to the KDIGO criteria. The risk factors for AKI were identified using a multivariate logistic regression model.

**Results:**

A total of 256 patients were included in this study, and the incidence of AKI was 18% (46/256). Patients in the AKI group were more likely to have a higher proportion of the youth, a higher level of body mass index, and a shorter time from onset to admission. Multivariate logistic regression analysis revealed that the youth (age ≤40 years) (OR: 2.853, 95%CI: 1.061–7.668, *p* = .038) were prone to AKI, and lower estimated glomerular filtration rate (eGFR) (OR: 1.526, per 15-ml/min/1.73 m^2^ decrease, 95%CI: 1.114–2.092; *p* = .009), higher diastolic blood pressure (DBP) (OR: 1.418, per 10-mmHg increase; 95%CI: 1.070–1.879; *p* = .015), and fasting blood glucose (FBG) ≥7 mmol/L on admission (OR: 2.592; 95%CI: 1.299–5.174; *p* = .007) were independent risk factors for AKI.

**Conclusions:**

Higher incidence of AKI had been perceived in this study, most of them were young and middle-aged patients. Renopreventive measures should be considered in those high-risk patients with younger age, lower eGFR, higher DBP, and higher FBG on admission.

## 1. Introduction

Aortic dissection (AD) is a catastrophic cardiovascular disease with high mortality [1], and acute kidney injury (AKI) is a frequent and serious complication of AD [2]. Convergent evidence from a multitude of studies shows that AKI is strongly associated with prognosis in AD patients [3–5]. Therefore, early identification and intervention are crucial for AKI prevention. The incidence of type A aortic dissection (TAAD) is higher than that of type B aortic dissection (TBAD), and TAAD is characterized by high mortality and poor prognosis in clinical practice [6]. Moreover, most TAAD patients, probably 86% reported by Pape et al. [7], require emergency surgery which may contribute to the occurrence of AKI [8,9]. As a result, most published studies focus on the perioperative AKI of TAAD patients, while the AKI of patients with TBAD, especially those with renal artery involvement, is rarely studied. The common risk factors for the occurrence of AKI in TAAD patients and TBAD patients include baseline renal function, blood pressure, renal artery involvement, etc. [3,10–12]. The risk factors of AKI for patients who underwent surgery for TAAD include perioperative sepsis, extracorporeal circulation time, and postoperative 72-h drainage volume, etc. [9,13,14].

Renal hypoperfusion occurs more frequently in TBAD patients than in those with TAAD [15], and renal artery involvement leads to a higher risk of AKI [16]. A study showed that the incidence of AKI was associated with DeBakey type IIIb (the dissection was extended below the diaphragm to involve the abdominal aorta in TBAD patients) [16]. The reason is that the pathologic states of TBAD involving renal arteries presents may influence renal function. And the rate of dysperfusion syndrome is greatly increased when the lesion involves the abdominal aorta and below, both blood supply of the collateral aorta from the false lumen (FL) and collateral artery occlusion due to free flap occlusion may affect renal perfusion [17]. This study aimed to identify the incidence and risk factors of AKI in TBAD patients with renal artery involvement and who received thoracic endovascular aortic repair (TEVAR) only.

## 2. Methods

### 2.1. Study population

All consecutive adult patients (≥18 years) who were diagnosed as TBAD and renal artery involvement in Beijing Anzhen Hospital, Capital Medical University (Beijing, China) between January 2015 and October 2019 were included in this study. All patients underwent TEVAR only and none of them had previously received renal replacement therapy. The patients’ medical records were collected and analyzed, including medical history, physical examination, laboratory measurements, and imaging examinations. Institutional review board approval was granted for the use of an aggregate data set for research and quality improvement by the Ethics Committee of Beijing Anzhen Hospital, Capital Medical University, and the principles outlined in the Declaration of Helsinki were followed. All participating patients signed informed consent.

### 2.2. Perioperative management

Patients presenting with renal insufficiency underwent preoperative hydration to make sure the urine output was over 100 mL/h. We gave the patients isotonic saline (1 mL/kg/h) from 12 h pre-procedure to 24 h post-procedure.

### 2.3. Description of procedure

Percutaneous TEVAR was conducted following standard procedures which began with the puncture of the common femoral arteries (CFAs) under fluoroscopy. 5 F or 6 F Perclose ProGlide devices were deployed in the CFA, and a platinum pigtail catheter was sent along the femoral artery to perform angiography of the abdominal aorta, descending thoracic aorta, and ascending aorta. After confirming the formation of Stanford TBAD and the location of the dissection lesion, the coated stent was transported along the hardened guidewire and released after precise positioning of the lesion. Aortography was performed again to confirm that the lesion had been successfully isolated.

All these patients use 100–120 mL contrast, and fluid resuscitation and vasoactive agents were give according to hemodynamics. Vasopressors mainly included epinephrine and norepinephrine, and antihypertensive drugs included nicardipine, isosorbide mononitrate, nitroglycerin, and esmolol.

### 2.4. Definitions

AKI was defined as an increase in serum creatinine (SCr) by ≥0.3 mg/dL (26.5 μmol/L) within 2 days or an increase in SCr to ≥1.5 times baseline within 7 days based on the Kidney Disease: Improving Global Outcomes (KDIGO) criteria [18]. Baseline creatinine was established using SCr previously recorded within 1 year or the lowest measurement during hospitalization [4]. Individuals with AKI were staged as follows: stage I as an increase in the SCr level by ≥0.3 mg/dL (26.5 μmol/L) or 1.5–1.9 times baseline; stage II as an increase in the SCr level by 2.0–2.9 times baseline; and stage III as an increase in the SCr level by ≥4.0 mg/dL (353.6 μmol/L) or ≥3.0 times baseline or the initiation of renal replacement therapy. Due to the lack of urine output data, we applied the creatinine criteria only. All patients were evaluated and divided into the AKI group and the non-AKI group. The estimated glomerular filtration rate (eGFR) was calculated from the first SCr value measured during hospitalization using epidemiology collaboration (EPI) equations [19].

All patients included in this study had undergone computed tomography angiography (CTA), and they were diagnosed as TBAD with renal artery involvement (the dissection was extended below the diaphragm to involve the abdominal aorta) according to Stanford classification [20]. Based on the imaging findings of CTA, all patients were divided into three groups: Type A, both renal perfusion were supplied from the aortic true lumen (TL); Type B: one side of renal perfusion was supplied from the aortic TL, while the other side originated from the aortic FL; Type C: both renal perfusions were supplied from the aortic FL. All images were independently analyzed by two experienced radiologists.

### 2.5. Statistical analysis

Statistical analysis was carried out using the SPSS software version 21.0. The continuous variables were presented as the mean ± standard deviation (SD) or median (interquartile range), and inter-group comparisons were performed using independent *t*-test or Mann–Whitney *U*-test when the distribution and variance met the appropriate conditions. Categorical variables were expressed as frequency (percentage) and analyzed using the chi-squared test or Fisher’s exact test when needed. Variables with less than 20% data missing and found to be statistically significant (*p* < .05) between two groups were included in univariate analyses (unadjusted), then the variables that had statistical differences (*p* < .05) were included in the multivariate logistic regression analysis. Odds ratios (OR), with 95% confidence intervals (CI) and *p*-value, were reported. Statistical significance was defined as a *p* < .05.

## 3. Results

### 3.1. Baseline characteristics

A total of 256 patients were included in the present study, 46 (18%) of them developed AKI. In those 46 patients who develop AKI, 12 (26.1%) of them had AKI before TEVAR, and 34 (73.9%) of them developed AKI after the procedure, including 39 (15.2%) in stage I, 4 (1.6%) in stage II, and 3 (1.2%) in stage III. The mean age of the patients was 52.8 ± 9.8 years, 217 (84.9%) were male, 228 (89%) were young and middle-aged people (age ≤65 years), 201 (78.8%) had a history of hypertension, and the median time elapsed from symptom onset to hospital admission was 7 days (1–19). Compared to the non-AKI group, patients in the AKI group were more likely to have a higher proportion of the youth, have a higher level of body mass index (BMI), shorter time from onset to admission. Moreover, the eGFR on admission of patients in the AKI group was significantly lower than that of the non-AKI group (79.8 ± 28.5 mL/min/1.73m^2^ vs. 90.9 ± 19.9 mL/min/1.73 m^2^, *p* = .022), while diastolic blood pressure (DBP) and fasting blood glucose (FBG) on admission in AKI group were higher (83.4 ± 13.0 mmHg vs. 79.1 ± 11.0 mmHg, *p* = .022; 7.4 ± 2.2 mmol/L vs. 6.8 ± 2.2 mmol/L, *p* = .025). However, no statistically significant differences were observed in sex, history of hypertension, systolic blood pressure (SBP) on admission, and classification of renal arteries between the two groups (Table 1).

**Table 1.** Demographics and baseline clinical characteristics.

Variables	All group (*n* = 256)	AKI group (*n* = 46)	Non-AKI group (*n* = 210)	*p*
Male, *n* (%)	217 (84.9%)	41 (89.1%)	176 (83.8%)	.363
Age, years, mean ± SD	52.8 ± 9.8	49.5 ± 10.1	53.5 ± 9.6	.012
Youth (age ≤40 years), *n* (%)	27 (10.5%)	9 (21.4%)	18 (9.7%)	.033
Young and middle-aged (age ≤65 years), *n* (%)	228 (89.1%)	42 (91.3%)	186 (88.6%)	.591
BMI, kg/m^2^, mean ± SD	27.2 ± 4.0	28.8 ± 4.6	26.8 ± 3.7	.021
History of hypertension, *n* (%)	201 (78.8%)	36 (78.3%)	165 (78.9%)	.918
CKD, *n* (%)	8 (3.1%)	4 (8.7%)	4 (1.9%)	.017
DM, *n* (%)	11 (4.3%)	0 (0%)	11 (5.2%)	.113
CHD, *n* (%)	16 (6.3%)	1 (2.2%)	15 (7.1%)	.207
History of ischemic stroke, *n* (%)	4 (1.6%)	1 (2.2%)	3 (1.4%)	.712
Cerebral hemorrhage, *n* (%)	9 (3.5%)	0 (0%)	9 (4.3%)	.153
Time from onset to admission, days, median (IQR)	7 (1, 19)	2 (1, 14)	7 (2, 20)	.013
SBP on admission, mmHg, mean ± SD	136.7 ± 19.9	143.1 ± 25.4	135.4 ± 18.2	.190
DBP on admission, mmHg, mean ± SD	79.9 ± 11.5	83.4 ± 13.0	79.1 ± 11.0	.022
SCr on admission, μmol/L, days, median (IQR)	77.7 (65.2, 92.7)	89.9 (73.2, 127.7)	76.5 (64.6, 90.6)	.002
eGFR on admission, mL/min/1.73 m^2^, mean ± SD	88.9 ± 22.1	79.8 ± 28.5	90.9 ± 19.9	.022
FBG on admission, mmol/L, mean ± SD	6.9 ± 2.2	7.4 ± 2.2	6.8 ± 2.2	.025
FBG on admission ≥7 mmol/L, *n* (%)	99 (39.3%)	73 (35.4%)	26 (56.5%)	.008
HGB on admission, g/L, median (IQR)	137 (124, 148)	142 (119, 152)	136 (125, 148)	.418
Classification				.355
Both TL, *n* (%)	92 (35.9%)	78 (37.1%)	14 (30.4%)	
TL and FL, *n* (%)	159 (62.1%)	127 (60.5%)	32 (69.6%)	
Both FL, *n* (%)	5 (2.0%)	5 (2.4%)	0 (0.0%)	
LVEF, %, mean ± SD	63 (60, 66)	62 (60, 71)	63 (60, 66)	.919
ASA classification (1/2/3/4), *n* (%)	0 (0%) / 38 (15.6%) / 196 (80.3%) / 10 (4.1%)	0 (0%) / 6 (13.0%) / 38 (82.6%) / 2 (4.3%)	0 (0%) / 32 (16.2%) / 158 (79.8%) / 8 (4.0%)	.870
Preoperative SBP, mmHg, median (IQR)	165 (150, 180)	160 (148, 176)	165 (148, 180)	.448
Preoperative DBP, mmHg, median (IQR)	80 (70, 85)	70 (65, 80)	80 (70, 85)	.100
Preoperative HR, beat/min, median (IQR)	70 (60, 80)	73 (64, 80)	70 (60, 80)	.248
Vasoactive medications, *n* (%)	181 (72.1%)	32 (69.6%)	149 (72.7%)	.670
Fluid infusion volume, mL, median (IQR)	500 (500, 750)	500 (500, 763)	500 (500, 750)	.273
Blood loss, mL, median (IQR)	0 (0, 20)	10 (0, 28)	0 (0, 20)	.216
blood transfusion, *n* (%)	3 (1.2%)	1 (2.2%)	2 (1.0%)	.505
OT, mL, median (IQR)	110 (80, 130)	110 (70, 143)	110 (100, 117)	.918
Endoleak, *n* (%)	3 (1.17%)	1 (2.2%)	2 (1.0%)	.486
Hospital stay, days, median (IQR)	8.0 (5.0, 11.0)	8.5 (6.0, 12.0)	7.0 (5.0, 15.0)	.089

AKI: acute kidney injury; BMI: body mass index; CHD: coronary heart diseases; CKD: chronic kidney disease; DBP: diastolic blood pressure; DM: diabetes mellitus; eGFR: estimated glomerular filtration rate; FBG: fasting blood glucose; FL: false lumen; HGB: Hemoglobin; HR: heart rate; IQR: interquartile range; LVEF: left ventricular ejection fraction; OT: operation time; SBP: systolic blood pressure; SD: standard deviation; SCr: serum creatinine; TL: true lumen.

None of them die during the hospitalization. As for complications related to TEVAR, 3 of them developed type Ia endoleak, and none of them need device migration or re-operation during the hospital stay. However, a few of them may need to re-operation because of the stent-graft induce new entry during the follow-up.

### 3.2. Risk factors of AKI

Univariate analysis (unadjusted) was carried out for all variables which had statistical differences except for BMI (absence of effective case number >20%), including the youth, eGFR, DBP, and FBG. Multivariate logistic regression analysis revealed that the youth (age ≤40 years) were prone to AKI, other risk factors for AKI in TBAD patients involving the renal artery including lower eGFR, higher DBP and FBG ≥ 7 mmol/L on admission (Table 2).

**Table 2.** Multivariate logistic regression analysis of risk factors for AKI.

Variables	Unadjusted		Adjusted	
	OR (95%CI)	*p*	OR (95%CI)	*p*
Youth (age ≤40 years old)	2.595 (1.083–6.219)	.033	2.853 (1.061–7.668)	.038
Time from onset to admission (days)	1.000 (0.998–1.001)	.555		
eGFR on admission (per 15-mL/min/1.73 m^2^ decrease)	1.407 (1.038–1.908)	.028	1.526 (1.114–2.092)	.009
DBP on admission (per 10-mmHg increase)	1.327 (1.020–1.726)	.035	1.418 (1.070–1.879)	.015
FBG on admission ≥7 mmol/L	2.250 (1.167–4.337)	.015	2.592 (1.299–5.174)	.007

AKI: acute kidney injury; DBP: diastolic blood pressure; DBP: diastolic blood pressure; eGFR: estimated glomerular filtration rate; FBG: fasting blood glucose; OR: odds ratios.

## 4. Discussion

AKI is a common and serious complication of TBAD, and it is associated with prolonged hospital stay duration and reduced survival [4]. AKI may progress to chronic kidney disease (CKD) or even end-stage renal disease (ESRD) if it was not diagnosed and managed promptly, which greatly increases the medical and financial burden on patients, families, and society [21,22]. Therefore, the prevention of AKI and frequent monitoring of renal function are vital for high-risk groups [23]. We compiled data for 256 TBAD patients involving the renal artery and treated by TEVAR only, most of them were young and middle-aged patients (89%), and the result showed that the overall incidence of in-hospital AKI in our study was 18%. The percentage of TBAD patients treated with open surgery was decreased dramatically in recent years (the rate presented by Evangelista was from 17% to 8% [6]), thus few studies focus on TBAD patients who received open surgery. A large retrospective study reported the incidence of AKI at 52.7% in TBAD patients, and the treatment of the study population included fenestration, branch vessel stenting, open surgery, and TEVAR [4]. Other previous studies, however, had variably reported the incidence of AKI among TBAD patients who were treated with either medication or TEVAR between 27.5% and 39.8% [10,16,17,24,25]. The present study showed a lower incidence of AKI compared to the above-mentioned studies. The possible reasons are shown as follows. Firstly, the study population varies among studies. Patients in this study had a longer disease duration than those in previous studies. Besides, most of them were young and middle-aged adults who were characterized by good health conditions. The physical function of the elderly is seriously reduced, and the function of the important organs is reduced. Besides, patients younger than 65 years old have lower rates of underlying diseases, in young and middle-aged TBAD patients of this study, 7 (3.1%) had CKD, 8 (3.5%) had DM, 10 (4.4%) had coronary heart disease, 2 (0.9%) had a previous cerebral hemorrhage, and 6 (2.6%) had a previous cerebral stroke. As a result, the operation tolerance of the aged people is significantly reduced. Furthermore, our population only received TEVAR, so they were not affected by the extracorporeal circulation or other serious complications during the perioperative period which is strongly associated with in-hospital AKI. And the lack of urinary output data also contributing to the lower incidence of AKI, because that making a diagnosis of AKI only with changes in creatinine level without urine output of the patient will cause the diagnosis of AKI to be missed in the group of patients who meet AKI criteria because of the decreased urine output, but whose creatinine value has not yet increased.

We studied the risk factors of AKI in TBAD patients with renal artery involvement. Multivariate analysis revealed that the youth, lower eGFR, higher DBP, and higher FBG on admission were independent risk factors for AKI. Several clinical risk factors for AKI were defined in previous studies of TBAD, including SCr on admission, SBP on admission, CKD, coronary heart disease, congestive heart failure, and history of hypertension [4,10,16,17,24,25]. Preexisting renal insufficiency is a well-recognized clinical risk factor of AKI, which still holds in many studies on TBAD [13,16,26]. Regarding the correlation between age and AKI, Wang et al. [14] reported that advanced age was an independent risk factor of AKI in a meta-analysis of seven published studies. However, our study showed that young people were a high-risk group of AKI, and there were insufficient reasons to say the results are opposite. The reason may be multifold. Firstly, the patients included in the above studies were diagnosed with TAAD and treated by open surgery. Furthermore, there were six articles included in the above-mentioned meta-analysis that reported average age, and the mean age of five studies was higher than this study. Generally, elderly patients with multiple chronic diseases or poor general health suffered a higher risk of AKI associated with open surgery than young and middle-aged patients [27]. TBAD patients in our study received TEVAR only, and most of them were young and middle-aged people (mean age of all cases in this study was 52.8 years), while only 7 (2.7%) patients were older than 70 years. Besides, in lots of previous studies, it had been confirmed that both a significant increase in blood pressure and long-term hypertension status are closely related to AKI [16,28]. Hypertension in young and middle-aged people is characterized by low awareness, treatment, and control rate, and they not only work or live in stressful environments but also have unhealthy lifestyle habits. The youth were a high-risk group of AKI, for the above characteristics were more significant in the youth than that of middle-aged patients [29]. We also found that DBP on admission was highly related to in-hospital AKI, perhaps because the young and middle-aged people are more likely to be isolated diastolic hypertension. Furthermore, FBG on admission was also one of the clinical risk factors which have been confirmed by previous studies [30–32]. Mechanisms might be oxidative stress, the promotion of inflammatory response, the activation of the renin–angiotensin system [31,32], and renal hypoperfusion induced by osmotic diuretic [33].

In recent years, several studies explored the association between the type of renal arteries involvement based on CTA (classification methods were not identical) in TBAD patients receiving TEVAR or drug treatment [10,16,17] (Table 3). Takahashi et al. [16] retrospectively analyzed the data of 56 TBAD patients treated by medications only and found that type IIIb compiling with narrowing or occlusion of one or both renal arteries diagnosed by CTA, history of hypertension, SCr on admission, and ST-T changes on ECG were risk factors for AKI. Ren et al. [10] reviewed 76 TBAD patients who underwent TEVAR only and reported the correlation between AKI and the arterial stenosis or thrombosis of bilateral renal arteries or SBP on admission. Besides, clinical data of 305 patients with TBAD and treated by TEVAR (12.5% of them suffered from supra-aortic branches graft bypass hybrid surgery) was retrospectively reviewed by Luo et al. [17] in 2017. They reported that SBP on admission and supra-aortic branches graft bypass hybrid surgery were closely related to AKI, but they didn’t provide enough evidence to figure out the association between AKI and the classification of renal arteries which is similar to this study. There are a couple of reasons for this. Firstly, CTA is a reliable imaging examination in detecting anatomical structures of renal arteries. However, it is considered to be imperfect in objectively predicting renal hypoperfusion because of the insufficient sensitivity [34], which can only identify static obstruction because the imaging just captures an arbitrary point in the cardiac cycle [15,34,35]. Besides, the actual perfusion of the renal artery may be more complicated. Digital subtraction angiography (DSA), which can assess renal blood flow and achieve a better understanding of dynamic obstruction, may solve this problem, which needs to be studied further. Distinct from the studies mentioned above, this study only recruited TBAD patients involving the renal artery, and most of them are young and middle-aged patients, so it has a guiding significance for preventing AKI in clinical practice, especially for young patients who are high-risk groups of AKI.

**Table 3.** Researches on AKI of TBAD patients.

	Present study	Study 1	Study 2	Study 3
First author, year of publication		Takahashi, 2014	Ren, 2015	Luo, 2017
Total number of patients	256	56	76	305
Renal artery involvement, *n* (%)	256 (100)	40 (71.4)	NR	NR
Age, years (mean)	53	64	51	55
Treatment	TEVAR	Medical therapy	TEAVR	TEAVR
Open surgery, *n* (%)	No	No	No	A part of (12.5)
AKI criteria	KDIGO	AKIN	KDIGO	KDIGO
Incidence of AKI (%)	18	36	36.8	27.5
CTA-based typing	Yes	Yes	Yes	Yes
Risk factors	eGFR on admission;	SCr on admission;	SBP on admission;	SBP on admission;
		Hypertension;		
	The youth (age ≤40 years); DBP on admission; FBG on admission	ST-T changes on ECG; Narrowing or occlusion of one or both renal arteries	Stenosis or thrombosis of bilateral renal arteries	Supra-aortic branches graft bypass hybrid surgery

AKI: acute kidney injury; AKIN: AKI Network; CTA: computed tomography angiography; DBP: diastolic blood pressure; eGFR: estimated glomerular filtration rate; FBG: fasting blood glucose; FL: false lumen; KDIGO: Kidney Disease Improving Global Outcomes; NR: not report; SBP: systolic blood pressure; SCr: serum creatinine; TBAD: type B aortic dissection; TEVAR: thoracic endovascular aortic repair.

There were a few limitations to our study. First, this was a retrospective single-center study, therefore, selection bias and confounding bias were unavoidable. Besides, the diagnosis of AKI was based on SCr value only, for the urine output data was missing in those hospitalized patients with urgent and serious conditions, which may underestimate the rate of AKI. Meanwhile, the present study could provide reliable evidence for randomized controlled trials controlled trial which a have more scientific clinical design.

## 5. Conclusions

In summary, the high incidence of in-hospital AKI had been perceived in TBAD patients with renal artery involvement, most of them were young and middle-aged people, and the youth were prone to AKI. Our study provided evidence that eGFR, DBP, and FBG on admission were significant independent risk factors of AKI. In TBAD patients, especially young patients or those who have lower eGFR, higher DBP, and higher FBG on admission need more attention. Therefore, the preliminary assessment of AKI and active management for the high-risk groups are essential to improve the prognosis of TBAD by preventing AKI. However, once the AKI has occurred, prompt treatment is critical for improving survival.
